# D2-Like Receptors Mediate Dopamine-Inhibited Insulin Secretion via Ion Channels in Rat Pancreatic β-Cells

**DOI:** 10.3389/fendo.2020.00152

**Published:** 2020-04-07

**Authors:** Mengmeng Liu, Lele Ren, Xiangqin Zhong, Yaqin Ding, Tao Liu, Zhihong Liu, Xiaohua Yang, Lijuan Cui, Lijun Yang, Yanying Fan, Yunfeng Liu, Yi Zhang

**Affiliations:** ^1^Department of Pharmacology, Shanxi Medical University, Taiyuan, China; ^2^Key Laboratory of Cellular Physiology, Ministry of Education, Shanxi Medical University, Taiyuan, China; ^3^Department of Endocrinology, The First Hospital of Shanxi Medical University, Shanxi Medical University, Taiyuan, China

**Keywords:** dopamine, D2-like receptors, insulin secretion, K_**V**_ channels, Ca^2+^ channels, β cells

## Abstract

Dopamine (DA) has a vital role in the central nervous system and also modulates lipid and glucose metabolism. The present study aimed to investigate the effect of dopamine on insulin secretion and the underlying mechanisms in rat pancreatic β-cells. Data from the radioimmunoassay indicated that dopamine inhibited insulin secretion in a glucose- and dose-dependent manner. This inhibitory effect of dopamine was mediated mainly by D2-like receptors, but not D1-like receptors. Whole-cell patch-clamp recordings showed that dopamine decreased voltage-dependent Ca^2+^ channel currents, which could be reversed by inhibition of the D2-like receptor. Dopamine increased voltage-dependent potassium (K_V_) channel currents and shortened action potential duration, which was antagonized by inhibition of D2-like receptors. Further experiments showed that D2-like receptor activation by quinpirole increased K_V_ channel currents. In addition, using calcium imaging techniques, we found that dopamine reduced intracellular Ca^2+^ concentration, which was also reversed by D2-like receptor antagonists. Similarly, quinpirole was found to decrease intracellular Ca^2+^ levels. Taken together, these findings demonstrate that dopamine inhibits insulin secretion mainly by acting on D2-like receptors, inhibiting Ca^2+^ channels, and activating Kv channels. This process results in shortened action potential duration and decreased intracellular Ca^2+^ levels in β-cells. This work offers new insights into a glucose-dependent mechanism whereby dopamine regulates insulin secretion.

## Introduction

Insulin production and utilization are essential for maintaining glucose homeostasis. In pancreatic β-cells, high glucose concentrations increase the ATP/ADP ratio, which leads to the closure of ATP-sensitive potassium channels and depolarization of membrane potential. The membrane potential depolarization activates voltage-dependent calcium channels and then promotes Ca^2+^ influx as well as insulin release. However, it is worth noting that voltage-dependent potassium (K_V_) channels are also activated during the procedure, which suppresses Ca^2+^ influx and insulin release (1, 2).

Dopamine is the most abundant catecholamine neurotransmitter in the brain, which modulates neuronal activity to perform various functions in the central nervous system (3). Studies have shown that dopamine can also be synthesized and released in peripheral tissues such as the pancreas, kidneys, and lungs to exert non-neural functions (4–6). Dopamine signals through the G protein-coupled dopamine receptors (DRs) to affect biological activities. Five different DR subtypes (D1R–D5R) have been identified to date, with different receptors being grouped based on their intrinsic properties into either the D1-like (D1R and D5R) or D2-like (D2R–D4R) subgroups (7, 8).

Clinical studies have reported that patients with Parkinson's disease who were treated with the long-term dopamine precursor L-dopa had a significant reduction in glucose tolerance and even developed type 2 diabetes (9). In recent years, dopamine has been proposed to be important in regulating glucose-stimulated insulin secretion (10, 11). However, the characteristic effect of dopamine on insulin secretion and the underlying electrophysiological mechanism have not been extensively studied.

In the current study, we focused on the acute effects of dopamine on insulin secretion and its influence on ion channels in pancreatic β-cells. The results demonstrated that dopamine suppresses insulin secretion in a glucose-dependent manner by binding to D2-like receptors, inhibiting Ca^2+^ channels, activating Kv channels, shortening action potential duration, and decreasing intracellular calcium levels.

## Materials and Methods

### Reagents

The dopamine, D1-like receptor inhibitor (SCH23390, 10 μM), D2-like receptor antagonist (eticlopride, 10 μM), D1-like receptor agonist (SKF38393, 10 μM), and D2-like receptor agonist (quinpirole, 10 μM) used in this study were purchased from Sigma-Aldrich, USA.

### Animals

Male Wistar rats (Beijing Vital River Laboratory Animal Technology) weighing 200–260 g were housed in an environment with suitable temperature (25 ± 2°C), humidity (55–60%), and 12 h of light–dark cycles. The animals had free access to food and tap water. All experiments involving animals described below were approved by the Animal Care and Use Committee of Shanxi Medical University.

### Isolation and Culture of Rat Pancreatic Islets and Islet Cells

After rats were euthanized, the prepared collagenase P (1 mg/mL, Roche, Indianapolis, USA) was injected into the pancreas via the common bile duct. After the pancreas was digested for 11 min, the isolated islets were obtained by density gradient centrifugation with Histopaque™-1077 (Sigma-Aldrich, USA). Pancreatic β-cells were separated from islets by Dispase II (Roche, Indianapolis, USA) digestion. Isolated islets and islet β-cells were cultured in HyClone RPMI 1640 medium (Hyclone, Beijing, China) supplemented with 10% fetal bovine serum, 100 mg/mL streptomycin, 100 U/mL penicillin, and 11.1 mM glucose at 37°C in 5% CO_2_ humidified atmosphere.

### Insulin Secretion Assay

Rat pancreatic islets (5 islets/vial) were pre-incubated in Krebs Ringer bicarbonate-HEPES (KRBH) buffer with 2.8 mM glucose (Solarbio, Beijing, China) for 30 min. Subsequently, islets were incubated in KRBH buffer containing different concentrations of glucose and drugs for 30 min. The concentrations of insulin in the supernatant were determined by an Iodine [^125^I] Insulin Radioimmunoassay Kit (North Biological Technology Research Institute of Beijing). The islets in each vial were lysed with 70% acid-ethanol solution [Ethanol/water/HCl (vol/vol) 150:47:3] for measurement of total cellular insulin concentration. The KRBH buffer contained: 128.8 mM NaCl; 4.8 mM KCl; 2.5 mM CaCl_2_; 1.2 mM KH_2_PO_4_; 1.2 mM MgSO_4_; 5 mM NaHCO_3_; 10 mM HEPES; and 2% BSA (Solarboi, Beijing, China) at pH 7.4.

### Patch-Clamp Experiments

Islet cells were cultured on coverslips coated with cell-adherent reagent (Applygen Technologies Inc., Beijing, China) for 24 h (12). The whole-cell patch-clamp was carried out using an EPC-10 amplifier and the PULSE software (HEKA Electronik, Lambrecht, GER). The resistances of patch pipettes filled with pipette solution ranged between 4 and 7 MΩ by employing Narishige PP-830 micropipette puller (Narishige Co, Tokyo, Japan) and MICROFORGEMF-200 (World Precision Instruments Inc, USA). β-cells were confirmed based on their capacitance values > 7 pF (13).

The Ca^2+^ currents were elicited by 50 ms pulses from a holding potential of −70 mV to the test potentials ranging from −50 to 30 mV in 10 mV steps. We replaced Ca^2+^ with Ba^2+^ in the extracellular solution to eliminate calcium-dependent inactivation of voltage-gated calcium channels. The holding potential of −70 mV was applied to limit contamination by Na^+^ currents, the majority of which are inactivated at this holding potential. The intracellular solution consisted of 120 mM CsCl; 20 mM TEA (Tetraethylammonium chloride, Sigma-Aldrich, USA); 1 mM MgCl_2_; 0.05 mM EGTA; 10 mM HEPES; and 5 mM MgATP (pH 7.3 adjusted with CsOH). The extracellular solution was made of 100 mM NaCl; 20 mM TEA; 20 mM BaCl_2_; 4 mM CsCl; 1 mM MgCl_2_; 5 mM HEPES; and 3 mM glucose (pH 7.4 adjusted with NaOH).

In order to record voltage-dependent potassium (K_V_) channel currents, the cells were voltage-clamped at a holding potential of −70 mV and then shifted to the test potentials ranging from −70 to 80 mV (10 mV steps) within 400 ms. The intracellular solution included: 10 mM NaCl; 140 mM KCl; 10 mM HEPES; 0.05 mM EGTA; and 1 mM MgCl_2_ (pH 7.25 adjusted with KOH). The extracellular solution was composed of 141.9 mM NaCl; 5.6 mM KCl; 1.2 mM MgCl_2_; 11.1 mM glucose; and 5 mM HEPES (pH 7.4 with NaOH).

The action potentials of β-cells were elicited by 4 ms, 150 pA current injection in the current-clamp mode. Action potential duration was determined based on the difference of time from the initial action potential until the membrane potential returned to within 10 mV of the resting membrane potential, which was processed by PULSE software (14).

### The Measurement of Intracellular Calcium Levels

Islet cells were cultured on coverslips coated by adhesion reagent for 3 h before experiments. Cells were incubated in KRBH buffer containing 2.8 mM glucose and 2 μM Fura-2 AM (Dojindo Laboratories, Japan) for 30 min at 37°C. Cells were then washed twice with the buffers supplemented with 2.8 mM glucose. Intracellular Ca^2+^ activity was monitored at excitation wavelength 340 and 380 nm, emission wavelength 510 nm by MetaFluor 7.8 (Molecular Devices, USA) software, and Olympus IX71 inverted microscope. The change in intracellular Ca^2+^ concentration was displayed by the ratio of fluorescence intensity (F340/F380). The ratio of fluorescence intensity (F/F_0_, where F is the average fluorescence intensity for 100 s after reaching a plateau under different treatments and F_0_ is the initial fluorescence intensity of 2.8 mM glucose) was used to compare intracellular Ca^2+^ levels under different treatments (15, 16).

### Statistical Analysis

Data were presented as mean ± SEM. Statistical analysis was done by parametric tests, Student's *t*-tests, or one-way ANOVAs in Sigmaplot (version 12.5). The *post-hoc* tests were done using the LSD-t test. *P* < 0.05 was considered a statistically significant difference.

## Results

### Dopamine Inhibits Insulin Secretion in a Dose- and Glucose-Dependent Manner

**Figure 1:**
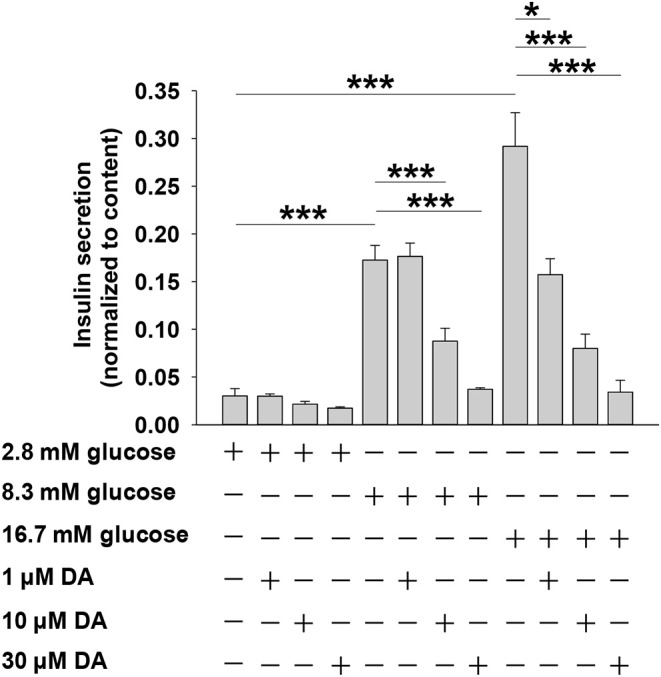
Dopamine (DA) inhibits insulin secretion in a dose- and glucose-dependent manner. Rat islets (5/vial) were treated with different concentration of DA in the presence of 2.8, 8.3, or 16.7 mM glucose. The supernatant was collected to test for insulin secretion. The islets in each vial were lysed with 70% acid-ethanol solution [ethanol/water/HCl (vol/vol) 150:47:3] for measurement of total cellular insulin content. Data are expressed as the mean ± SEM and compared by one-way ANOVA (*n* = 7 vials per group), *P < 0.05, ***P < 0.001.

We first investigated how dopamine affects insulin secretion in rat pancreatic islets. As shown in Figure 1, when islets were incubated with 2.8 mM glucose, dopamine had no effect on insulin secretion regardless of concentration (0–30 μM). However, at higher glucose conditions (8.3 or 16.7 mM), dopamine dose-dependently inhibited insulin secretion (Figure 1).

### Dopamine Inhibits Insulin Secretion Mainly via D2-Like Receptors, Rather Than D1-Like Receptors

**Figure 2:**
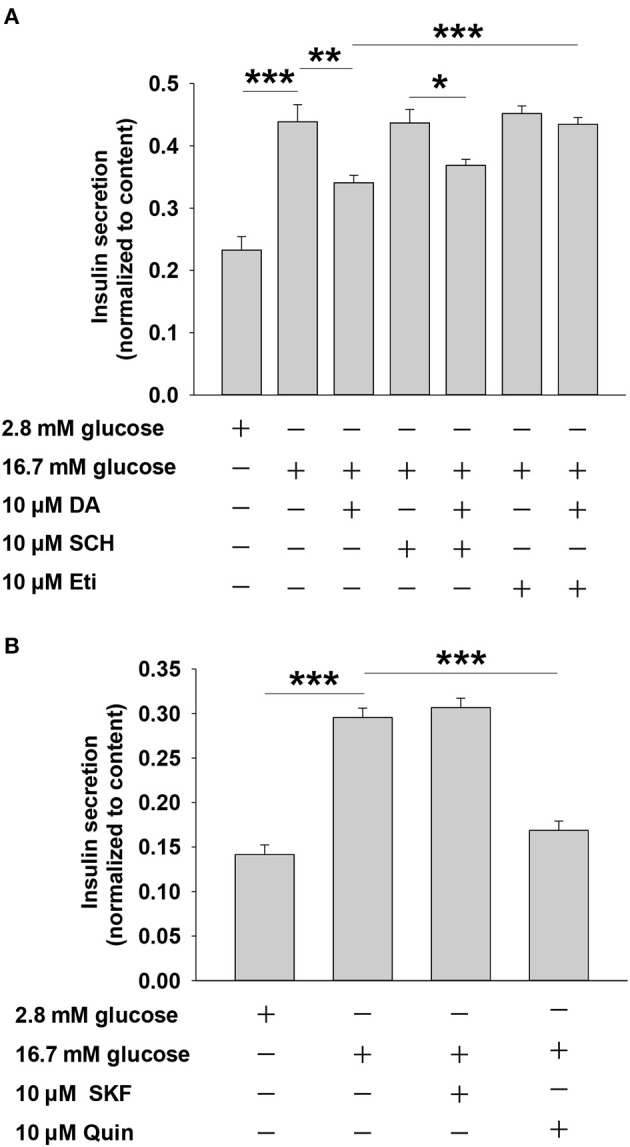
Dopamine (DA) inhibits insulin secretion mainly via D2-like receptors, rather than D1-like receptors. **(A)** Effect of SCH 23390 (SCH, 10 μM) and eticlopride (Eti, 10 μM) on DA-inhibited insulin secretion. **(B)** Effect of SKF38393 (SKF, 10 μM) and quinpirole (Quin, 10 μM) on DA-inhibited insulin secretion. Rat islets (5/vial) were incubated under different conditions. Insulin was determined by Iodine [^125^I] Insulin Radioimmunoassay Kit. Data are expressed as the mean ± SEM and compared by one-way ANOVA (*n* = 6 vials per group), *P < 0.05, **P < 0.01, ***P < 0.001. SCH 23390, D1-like receptor antagonist; eticlopride, D2-like receptor antagonist; SKF38393, D1-like receptor agonist; quinpirole, D2-like receptor agonist.

Dopamine exerts physiological effects through G protein-coupled receptors, including the D1-like receptor subfamily (D1 and D5) and the D2-like receptor subfamily (D2, D3, and D4) (8), which are expressed in rat islets (17). To investigate the relationship between dopamine receptors and dopamine-inhibited insulin secretion, we applied agonists or antagonists of the two dopamine receptor subfamilies. Eticlopride is a potent and selective D2-like receptor antagonist. Previous studies showed that eticlopride was more specific for D2-like receptors than pimozide or haloperidol (18). Quinpirole has been used as a selective D2-like receptor agonist in various experiments (17, 19). As shown in Figure 2A, the D2-like receptor antagonist eticlopride markedly blocked the effect of dopamine on insulin secretion, whereas SCH23390 (D1-like receptor inhibitor) had no influence on dopamine-inhibited insulin secretion in rat pancreatic islets. Furthermore, we found that the D2-like receptor agonist quinpirole decreased insulin secretion, while the D1-like receptor agonist SKF38393 had no effect on insulin secretion (Figure 2B). Together, these results indicate that D2-like receptors are the primary mediators of dopamine-inhibited insulin secretion from rat pancreatic islets.

### Dopamine Inhibits Voltage-Dependent Ca^2+^ Channel Through D2-Like Receptors

**Figure 3:**
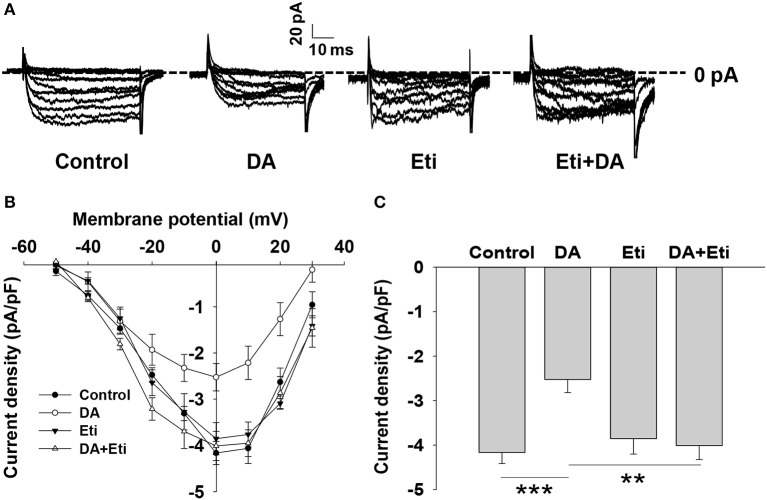
Dopamine reduces voltage-dependent calcium channel currents. **(A)** Representative Ca^2+^ current traces recorded from different rat β-cells treated with dopamine (DA, 10 μM) in the presence or absence of eticlopride (Eti, 10 μM). **(B)** Current-voltage relationship curves obtained from the treated cells. **(C)** Summary of the mean current density of Ca^2+^ currents at 0 mV depolarization. Data are expressed as the mean ± SEM and compared by one-way ANOVA. Pancreatic β-cells were confirmed based on their capacitance values >7 pF (*n* = 6 β-cells/group), **P < 0.01; ***P < 0.001.

It is well established that voltage-dependent Ca^2+^ channels play an important role in glucose-stimulated insulin secretion (20, 21). We examined the effect of dopamine on Ca^2+^ channels. The inward Ca^2+^ currents were evoked by depolarizing pulses with whole-cell patch-clamp technology (Figure 3A). Compared to the control, dopamine prominently decreased voltage-dependent Ca^2+^ currents (Figures 3A–C). Since D2-like receptors are required for dopamine-inhibited insulin secretion, we further tested the role of D2-like receptors in dopamine-regulated Ca^2+^ channels. As shown, eticlopride itself had no effect on voltage-dependent Ca^2+^ channels. However, the inhibition of Ca^2+^ channels by dopamine was abolished in the presence of eticlopride (Figures 3A–C). These results indicate that dopamine-regulated Ca^2+^ channels are mediated by D2-like receptors.

### Dopamine Increases Voltage-Dependent Potassium (K_V_) Channel Currents via D2-Like Receptors

**Figure 4:**
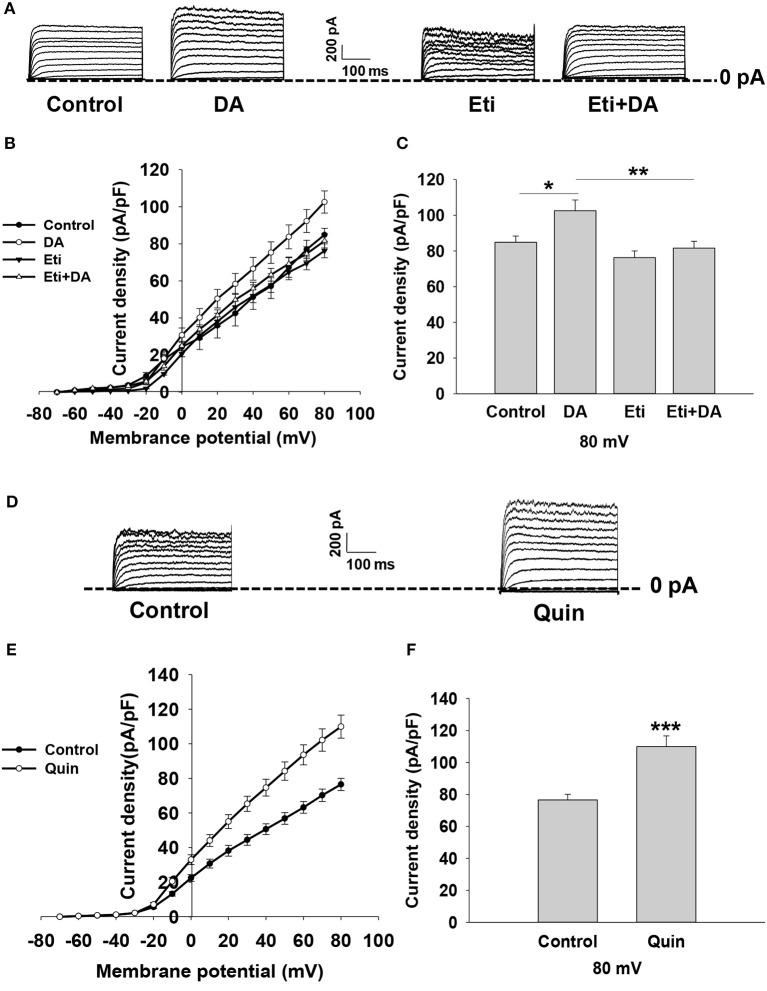
Dopamine increases K_V_ currents via D2-like receptors in rat β cells. K_V_ currents were recorded from a holding potential of −70 to 80 mV in 10 mV increments. **(A)** Representative whole-cell current traces recorded when different cells were treated with dopamine (DA, 10 μM) in the presence or absence of eticlopride (Eti, 10 μM). **(B,E)** Current-voltage relationship curves of Kv channels under different treatments. **(C,F)** Summary of the mean current density of Kv channels recorded at 80 mV depolarization. **(D)** Representative whole-cell current traces recorded in the presence or absence of quinpirole (Quin, 10 μM). Data are expressed as the mean ± SEM and compared by one-way ANOVA and Student's *t*-test. Pancreatic β-cells were confirmed based on their capacitance values >7 pF (*n* = 6 β-cells/group), *P < 0.05; **P < 0.01; ***P < 0.001.

Kv channels have been shown to play an important role in modulating stimulus–secretion coupling in pancreatic β-cells (22). We next examined the influence of dopamine on Kv channels in rat β-cells. Outward K^+^ currents were evoked by depolarizing pulses from potentials of −70 to 80 mV in whole-cell voltage-clamp experiments (Figure 4A). The data showed that dopamine significantly enhanced Kv currents compared to controls (Figures 4A–C), suggesting that Kv channels are involved in dopamine-inhibited insulin secretion.

To test whether dopamine-regulated Kv currents are mediated by D2-like receptors, pancreatic β-cells were treated with D2-like receptor antagonist eticlopride in the presence or absence of dopamine. We found that eticlopride alone did not influence Kv channels. However, eticlopride remarkably abolished the effect of dopamine on Kv channel currents (Figures 4A–C). We further applied D2-like receptor agonist quinpirole to verify the effect of D2-like receptors on Kv channels. As shown in Figures 4D–F, quinpirole significantly increased K_V_ currents compared to controls. Thus, these data indicate that dopamine increases Kv currents via D2-like receptors.

### Dopamine Shortens Action Potential Duration (APD) via D2-Like Receptors

**Figure 5:**
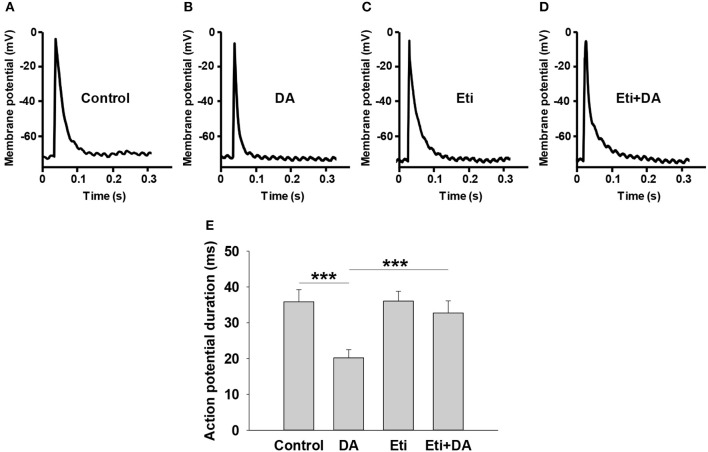
Dopamine shortens action potential duration via D2-like receptors. Action potentials were elicited from pancreatic β-cells in different groups by applying 4 ms, 150 pA current injection. The indicated drugs were added to the cell chamber with bath solution before patch-clamping until the experiment was finished. **(A–D)** Representative action potential waveforms obtained under different conditions as indicated. **(E)** Summary of the mean action potential durations. Data are expressed as the mean ± SEM and compared by one-way ANOVA (*n* = 6 β-cells/group), ***P < 0.001. Dopamine (DA, 10 μM); Eticlopride (Eti, 10 μM).

Glucose-stimulated insulin secretion is triggered by a series of electrical activity from pancreatic β-cells (23). Among them, the activation of Kv channels repolarizes action potentials to limit insulin secretion (24). We performed whole-cell current-clamp experiments to assess if dopamine affects action potential duration in rat pancreatic β-cells. As depicted in Figures 5A,B,E, dopamine notably shortened action potential durations compared to controls (*P* < 0.001).

We next explored the relationship between D2-like receptors and dopamine-regulated action potential duration (APD). As shown in Figures 5A,C,E, the D2-like receptor antagonist eticlopride had no influence on APD by itself. However, the effect of dopamine on APD was significantly abolished by eticlopride (Figures 5B,D,E), suggesting that D2-like receptors mediate dopamine-regulated APD.

### Dopamine Decreases Intracellular Calcium ([Ca^2+^]_i_) Level Through D2-Like Receptors in Rat Pancreatic β-Cells

**Figure 6:**
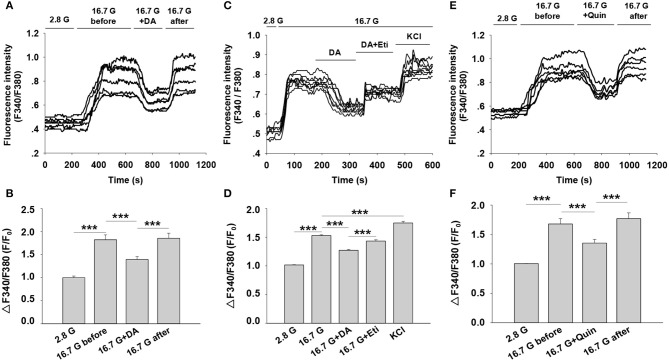
Effect of dopamine on intracellular calcium ([Ca^2+^]_i_) level in rat pancreatic β-cells. [Ca^2+^]_i_ level was determined based on the ratio of F340/F380. **(A)** Cells were perfused with dopamine (DA, 10 μM) in the presence of 16.7 mM glucose (16.7 G) (*n* = 6 β-cells/group). **(C)** Cells were treated with dopamine (DA, 10 μM) in the presence or absence of eticlopride (Eti, 10 μM) (*n* = 8 β-cells/group). **(E)** Cells were perfused with quinpirole (Quin, 10 μM) in the presence of 16.7 mM glucose (*n* = 6 β-cells/group) **(B,D,F)**. The mean of ΔF340/F380 (F/F_0_) under different treatments as indicated. F, average fluorescence intensity during 100 s after the fluorescence plateaued for different treatments; F_0_, baseline fluorescence at 2.8 mM glucose (2.8 G). Data are expressed as the mean ± SEM and compared by one-way ANOVA. ***P < 0.001.

Intracellular Ca^2+^ plays a pivotal role in insulin secretion. Glucose-induced electrical activity is accompanied by changes of intracellular Ca^2+^ level in pancreatic β-cells (23). We therefore examined the influence of dopamine on [Ca^2+^]_i_ level by applying Fura-2 AM dye. The change in [Ca^2+^]_i_ was reflected by the ratio of fluorescence intensity (F340/F380) in β-cells. As shown in Figures 6A,B, 16.7 mM of glucose significantly increased fluorescence intensity compared to 2.8 mM of glucose. However, dopamine markedly lowered fluorescence intensity under 16.7 mM glucose conditions, demonstrating that dopamine decreased [Ca^2+^]_i_ level in rat pancreatic β-cells.

To explore the influence of D2-like receptors on dopamine-regulated [Ca^2+^]_i_, we performed experiments with the D2-like receptors antagonist eticlopride. Figures 6C,D showed that the decreased fluorescence intensity induced by dopamine was counteracted by eticlopride. In addition, we found that the fluorescence intensity was also reduced by using the D2-like receptor agonist quinpirole under the 16.7 mM glucose condition (Figures 6E,F). These findings suggest that the action of dopamine on [Ca^2+^]_i_ is mediated by D2-like receptors in rat pancreatic β-cells.

## Discussion

Dopamine receptors have been detected in the islets of human, rat, mouse (25), and INS-1E β cells (17). In the present study, we found that dopamine and the D2-like receptor agonist quinpirole play similar roles in inhibiting insulin secretion from rat pancreatic islets, which is consistent with previous reports (17). Dopamine-inhibited insulin secretion was antagonized by the D2-like receptor blocker eticlopride. In addition, we found that applying the D1-like receptor agonist SKF38393 or the antagonist SCH 23390 had no significant effect on insulin secretion. These results demonstrate that dopamine inhibits insulin secretion mainly via D2-like receptors in rat pancreatic islets. Meanwhile, our data indicates that the characteristic of dopamine in regulating insulin is glucose-dependent, since dopamine exerts the inhibitory effect at high glucose concentrations (8.3 and 16.7 mM glucose) rather than at basal glucose concentration (2.8 mM glucose).

Dopamine influences ion channels such as voltage-gated K^+^, Na^+^, and Ca^2+^ channels in the central nervous system (26). In the large neuron, dopamine reduces the frequency of spontaneous action potentials and activates inward rectifier K^+^ channels (27). Moreover, dopamine decreases cytosolic Ca^2+^ concentration in rat lactotroph cells (28). Considering that insulin secretion from pancreatic β-cells is regulated principally by ion channels, we examined the potential electrophysiological mechanism of dopamine in regulating insulin secretion.

Our study showed that dopamine reduced voltage-dependent Ca^2+^ channel currents and increased Kv channel currents in β-cells via D2-like receptors. It is well-established that voltage-dependent Ca^2+^ channels are responsible for the generation of action potentials and serve as a positive regulator of insulin secretion (29). K_V_ channels are important for the repolarization of action potentials and act as a negative regulator of insulin secretion (24). Our data reveals that dopamine significantly shortens action potential duration, which appears to be the consequence of Ca^2+^ channel inhibition and K_V_ channel activation caused by dopamine.

Intracellular calcium ([Ca^2+^]_i_) is a key determinant of glucose-stimulated insulin secretion (30, 31). As expected, our calcium imaging results showed that dopamine reversibly decreased [Ca^2+^]_i_ via D2-like receptors in rat pancreatic β-cells, which is consistent with the results of our secretion assay. Our study reveals that the reduction of [Ca^2+^]_i_ is due to dopamine inhibition on Ca^2+^ channels. Furthermore, because activation of K_V_ channels shortens action potential durations to limit total Ca^2+^ influx, we propose that the K_V_ channel activation also contributes to the reduction of [Ca^2+^]_i_ induced by dopamine.

Further, dopamine-regulated insulin secretion is found to be glucose-dependent, which could also be ascribed to the inhibition of Ca^2+^ channels and the activation of K_V_ channels, because the Ca^2+^ channels and K_V_ channels are only open in response to membrane depolarization under high glucose condition (32).

Studies have demonstrated that D2-like receptors are coupled to Gα_i/o_ proteins, leading to inhibition of adenylyl cyclase (AC) and protein kinase A (PKA) activity in neurons (33). Forskolin, an activator of cAMP, has been shown to antagonize the inhibitory effect of dopamine on insulin secretion (34). Furthermore, we and others have shown that AC and the downstream cAMP/PKA play an important role in glucose-stimulated insulin secretion, including a voltage-dependent ion channel mechanism (14, 35, 36). Given these findings, we speculate that the AC/cAMP/PKA signaling pathway might be involved in the regulation of dopamine on insulin release.

In the present study, Ca^2+^ channel current measurements and action potential recordings were carried out in non-physiological conditions, which helps identify their roles in the regulation of insulin secretion by dopamine. However, exploring the downstream signaling pathways and identifying the involvement of the master receptor of the D2-like family is also essential for a better understanding of the influence of dopamine on insulin secretion. A recent study using transgenic knockout mice reveals that both D2R and D3R mediate the inhibitory effect of dopamine on insulin secretion (37). Likewise, efforts are expected to further discern the metabolic roles and clinical implications of the dopaminergic system in pancreatic islets in future research.

In summary, these results collectively suggest that dopamine inhibition on insulin secretion is associated with the activation of D2-like receptors followed by inhibition of voltage-dependent Ca^2+^ channels and activation of K_V_ channels, which shortens glucose-stimulated action potentials and limits Ca^2+^ entry, leading to the suppression of insulin secretion. As a neurotransmitter in the central nervous system, dopamine has been shown to be important in the regulation of glucose homeostasis (38). Our findings provide new insight into dopamine-mediated regulation of insulin secretion in primary rat islets, which also explains the cause of impaired insulin secretion and impaired glucose tolerance in Parkinson's disease patients treated with long-term dopaminergic drugs.

## Data Availability Statement

The datasets generated for this study are available on request to the corresponding author.

## Ethics Statement

The animal study was reviewed and approved by The Animal Care and Use Committee of Shanxi Medical University.

## Author Contributions

All authors participated in the experimental design, data analysis, and interpretation. ML, LR, XZ, and YD performed the research and developed methods. YZ, ML, XZ, and LR designed the study. YZ, YL, and ML wrote the manuscript. TL, ZL, XY, LC, LY, YF, and YL critically read the manuscript and participated in fruitful discussions.

### Conflict of Interest

The authors declare that the research was conducted in the absence of any commercial or financial relationships that could be construed as a potential conflict of interest.
